# Development of Clinical Referral Score Model for Early Diagnosis of Hirschsprung’s Disease in Suspected Pediatric Patients

**DOI:** 10.3390/healthcare9060678

**Published:** 2021-06-04

**Authors:** Jiraporn Khorana, Phawinee Phiromkanchanasak, Jitthiwimon Kumsattra, Suparada Klinoun, Suthasinee Aksorn, Sireekarn Chantakhow, Kanokkan Tepmalai, Jesda Singhavejsakul

**Affiliations:** 1 Department of Surgery, Division of Pediatric Surgery, Faculty of Medicine, Chiang Mai University Hospital, Chiangmai 50200, Thailand; sireekarn.chan@cmu.ac.th (S.C.); kanokkan.t@cmu.ac.th (K.T.); jesda.s@cmu.ac.th (J.S.); 2 Center of Clinical Epidemiology and Clinical Statistic, Faculty of Medicine, Chiang Mai University Hospital, Chiangmai 50200, Thailand; 3 Faculty of Medicine, Chiang Mai University Hospital, Chiangmai 50200, Thailand; phawinee_phiromkan@cmu.ac.th (P.P.); jitthiwimon_k@cmu.ac.th (J.K.); suparada_k@cmu.ac.th (S.K.); suthasinee_aksorn@cmu.ac.th (S.A.)

**Keywords:** Hirschsprung’s disease, clinical diagnosis, contrast enema, rectal suction biopsy, pediatric surgery, pediatric emergency

## Abstract

The diagnosis of Hirschsprung’s disease (HSCR) relies on history, physical examination, and investigations. Some of investigation modalities could not be done in primary hospital. This study was aimed to develop the clinical score model for diagnosing and early referrals of HSCR, especially in areas where investigations were not available. Overall 483 consecutive suspected HSCR patients who were under 15 years old from January 2006 to December 2020 were included in this study, with 207 (42.86%) patients diagnosed with HSCR and 276 (51.14%) patients in the non-HSCR group. Five clinical parameters were included in the prediction model. The AuROC of clinical parameters, which included having an age younger than one month, male gender, the term infant, history of delayed meconium passage, and history of enterocolitis, was 72%. The prediction score ranged from 0–7, with a score 0–3 meaning a low risk to be HSCR (LHR+ = 0.37). We concluded that patients with suspected HSCR who had clinical score 4–7 had a high probability to be HSCR and, thus, it was suggested that these patients have an early referral for further investigations, which were contrast enema and rectal suction biopsy. In the case of a low probability of HSCR, clinical observation is still warranted. This clinical scoring system can be used as a screening tool to prevent delay diagnosis and complications.

## 1. Introduction

Hirschsprung’s disease (HSCR), or congenital aganglionic megacolon, occurs in approximately 1 in 5000 of all live-born infants. This disease occurrs because of the absence of the ganglion plexus in the large intestine, which causes the large bowel to lose its ability for dilation or peristalsis resulting in intestinal obstruction [1].

Patients with HSCR could develop obstructive symptoms days after birth such as the delayed passage of meconium (failure to pass first meconium within 48 h of life), abdominal distention, constipation, bilious vomiting, failure to thrive, and absence of flatus. Some studies [2,3] define common clinical manifestations as a “classic triad” including delayed passage of meconium, abdominal distention, and vomiting. Physical examination shows signs of malnutrition such as low height and weight. Increased rectal tone and explosive stool after rectal examination are characteristic of HSCR. In some cases, HSCR could have severe complications such as Hirschsprung-associated enterocolitis, resulting in intestinal perforation and sepsis as a presentation.

The diagnosis of HSCR comprised of clinical presentations, physical examination together with other investigations such as abdominal plain film, contrast enema, anorectal manometry, and a rectal biopsy. A contrast enema is commonly used for initiating investigations in HSCR-suspected patients due to its high sensitivity and its availability in most healthcare centers. However, there are various sensitivities and specificities of contrast enema in other studies; thus, further investigations must be performed to give a diagnosis [4,5,6]. Anorectal manometry usually needs special instrument to be performed. The sensitivity and specificity are also not as high as a rectal biopsy.

Rectal biopsy is the most specific and representative in diagnosis of HSCR. Two biopsy techniques are commonly performed. The first one is a full-thickness rectal biopsy, which could be performed in a surgical setting and is an acceptable reference standard test to diagnose HSCR. The second newer and less invasive technique, rectal suction biopsy (RSB), can be interpreted if there is an adequate specimen. According to the European Reference Network for rare inherited and congenital digestive disorders (ERNICA) guideline, rectal histology is required for diagnosis. Both techniques are accurate if adequate submucosal tissue is included [7]. The less invasive procedure, which is rectal suction biopsy, should be considered first. A diagnosis of HSCR is given if the specimen shows an absence of ganglion cells in the hematoxylin and eosin stain or a special immunohistochemistry study, such as acetylcholine esterase or calretinin stain, which shows an aganglionic segment [8]. Some studies [3,9] proposed performing a rectal biopsy in patients with signs of abdominal obstruction including the following: delayed passage of meconium, abdominal distention, and vomiting, along with positive findings from other investigations to avoid unnecessary invasive procedures. On the other hand, some studies [10,11] recommended performing a rectal biopsy in every patient with suspected HSCR despite a high rate of negative results, because many HSCR patients presented with only one symptom and late diagnosis could increase the risk of developing life-threatening complications.

However, without the results from a rectal biopsy, it could be difficult to diagnose HSCR, because some intestinal obstructive conditions manifest similarly to HSCR, such as meconium plug syndrome, anorectal malformation, malrotation, intestinal atresia, and meconium ileus associated with cystic fibrosis [2]. In addition, some non-obstructive conditions, such as allergic proctitis [12,13], lactase deficiency, celiac disease [2,13,14], cow’s milk allergy [2,13], hypothyroidism [2,15], and enterocolitis, could also mimic HSCR.

The other group of conditions, called variants or allied disorders of Hirschsprung’s disease [16], show a close resemblance to HSCR in terms of clinical presentations and findings from the investigations, although the ganglion cells are present in the biopsy. The examples are intestinal neuronal dysplasia, intestinal ganglioneuromatosis, isolated hypoganglionosis, immature ganglia, absence of the argyrophilic plexus, internal anal sphincter achalasia, and megacystis microcolon intestinal hypoperistalsis syndrome.

In our study, the diagnosis of HSCR was still challenging in the remote hospital with no complete investigation options. There was no specific protocol to refer suspected HSCR patients for further investigation. Either delayed diagnosis or over-investigation could occur. The mode of diagnosis was comprised of clinical evaluation, plain abdominal radiography, contrast enema, and rectal biopsy. This study aimed to help the remote hospital to screen the clinical presentation for diagnosing HSCR and for the early referral of the suspected patient to the center that could perform further investigation.

## 2. Materials and Methods

This study was designed as a retrospective cohort study with approval from the Research Ethics Committee, Faculty of Medicine, Chiang Mai University (Study code: SUR-2563-07500) with an exemption from patient informed consent due to the full retrospective study.

### 2.1. Participants

The data were collected from patients with suspected HSCR in Chiang Mai University Hospital that were presented from 2006 to 2020 and recorded in the database. Clinically suspected HSCR in this study included abdominal distension, constipation, vomiting, or plain abdominal radiography that showed large bowel dilation with no gas presented in rectum. The participants included patients with clinically suspected HSCR less than 15 years old at the date of consultation. The participants needed to have at least one clinical presentation suggesting HSCR and underwent either contrast enema, rectal biopsy, or curative surgery.

Patients diagnosed with HSCR with a colostomy from the previous hospital lacked initial clinical presentations of HSCR; thus, these patients were excluded from this study. Patients with an anorectal malformation, which is congenital anatomical anomalies that also cause bowel obstruction symptoms, except for anal stenosis, were excluded from this study because HSCR could simply be ruled out by physical examination. Patients lost to follow-up during the procedure were also excluded due to an unknown final diagnosis.

### 2.2. Outcome

The participants were diagnosed with HSCR if results from operative specimens, assessed by experienced pathologists, showed the absence of ganglion cells in the distal bowel. The non-Hirschsprung’s disease group (non-HSCR) consisted of participants whose ganglion cells were present or calretinin positive in the rectal biopsy specimen with no recurrence of clinical presentations after six months of follow-up. Some participants who underwent surgery in the early years of the study and had ganglion cells present in operative specimens were included in the non-HSCR group because of inadequate evidence to be HSCR.

### 2.3. Predictors

Parameters recorded in this study were categorized into the following three groups: general information, clinical presentations, and physical examinations.

The general information group consisted of age, prematurity, gender, and Down’s syndrome. Participants defined as term had a gestational age more than or equal 37 weeks [17].

Clinical presentations of patients included the history of delayed passage of meconium at 48 h, abdominal distention, constipation, history of enterocolitis, and vomiting.

The physical examination group consisted of weight and results of per-rectal examinations including increased rectal sphincter tone and explosive stool after the examination. Weight was converted to weight-for-age percentile according to the World Health Organization growth standard [18]. Failure to thrive was defined by the patient’s weight being less than the 5th weight-for-age percentile [19]. However, the cut-off point of the weight-for-age percentile in the predictive models was determined later on the basis of the power of prediction.

### 2.4. Sample Size

The sample size was calculated on the basis of the sensitivity of the statistically significant parameter, which was 81.1% of the parameter age less than three years old, as provided from the previous study [20]. With the significance level (α) of 0.05 and the maximum marginal error (d) of 0.05, the number of approximate sample sizes in this study was 421.

### 2.5. Missing Data

The multiple imputation methods were used for the imputation of the missing listed parameters, which were term, history of delayed passage of meconium at 48 h, and history of enterocolitis (missing at 102, 175, and 300 out of 483 participants, respectively).

### 2.6. Additional Data

The additional investigations consisted of results from contrast enema, rectal suction biopsy, and full-thickness biopsy specimen. As anorectal manometry was only recently available in the institution, the result of anorectal manometry was not included in this study. Transitional zone [21], jejunization, saw-tooth appearance, reverse rectosigmoid ratio (<1), and delayed evacuation in 24 h were collected as contrast enema signs. The contrast enema result was assessed by a radiologist to determine whether the results suggested HSCR. The outcome of the rectal suction biopsy was pathologic results with the addition of calretinin marker. In addition, some of the investigations were not performed but the data were also included in this study. This information was added in this study to compare the result with the developmental predictive model.

### 2.7. Statistical Analysis

Statistical analysis was performed with commercial statistical software (STATA 16.0; StataCorp LP, College Station, TX, USA). Categorical descriptive statistics were presented by count and percentage. Continuous descriptive statistics were presented by the mean and standard deviation or median and interquartile range (IQR) according to data distribution. Categorical analytic statistics were calculated from Fisher’s exact test. Continuous analytic statistics were calculated from Student’s *t*-test or Mann–Whitney U Test. The significance level in this study was 0.05.

Multivariable analysis was done by logistic regression reported by diagnostic odds ratio (dOR). Various combinations of significant clinical parameters listed above that had a univariable *p*-value < 0.01 were selected in the model and removed by the stepwise method to retrieve all significant predictive parameters in the developed model.

Total of 5 parameters were used in the reduced model. The assigned score was done by transforming the regression coefficient of dOR. Total score of 1–7 was derived from the model with the cut-off point of 4 to achieve the high true positive rate (sensitivity) to promote active referrals of the suspected patient. The likelihood ratio of positive values for diagnosing HSCR was presented.

The receiver operating characteristic (ROC) curve, and area under the ROC curve (AuROC) were calculated to derive the discriminative potential of the model. Hosmer–Lemeshow goodness of fit statistics and a calibration plot comparing the agreement of the observed and expected score values were also presented. The bootstrapping procedure with 200 replicates was executed for internal validation of the model.

The diagnostic indices of the predictive model, contrast enema, and rectal suction biopsy were calculated including sensitivity, specificity, negative predictive value (NPV), and positive predictive value (PPV). The AuROC of the combination of the model with the investigation was presented.

## 3. Results

**Figure 1:**
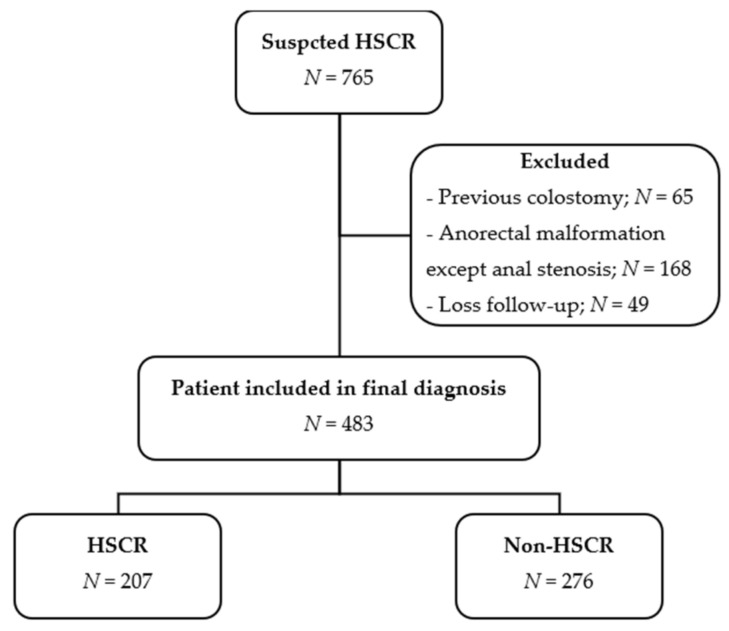
Study flow diagram.

First, data on 765 participants with suspected HSCR were collected. Then, 483 were included in the final analysis, as shown in Figure 1. Of the included participants, 194 (40.2%) were female and 289 (59.8%) were male. The median age of presentation was 37 days (IQR = 20.0–112.0). Overall, 314 out of 381 (82.4%) patients were term. Additionally, 13 (3.7%) out of 349 participants had down’s syndrome.

The participants were divided into two groups including 207 (42.86%) patients diagnosed as HSCR and 276 (51.14%) patients categorized in the non-HSCR group. There were seven patients diagnosed with total colonic aganglionosis within the HSCR group.

In the non-HSCR group, definitive diagnoses were identified in 110 out of 276 participants. The majority were constipation (63, 57.3%), anal stenosis (9, 8.2%), enterocolitis (5, 4.5%), hypothyroidism (2, 1.8%), and other diagnoses (31, 28.2%). Some other diagnoses identified were meconium plug syndrome, intestinal atresia, lactase deficiency, malrotation, inguinal or umbilical hernia, and overfeeding. The remainder had an uncertain diagnosis.

The general characteristics of the participants were shown in Table 1. The parameters that showed univariable statistical significance for diagnosing HSCR were age, term, percentile weight for age, male gender, and history of delayed passage of meconium. The number of the participants that provided the univariable data are also presented in Table 1.

**Figure 2:**
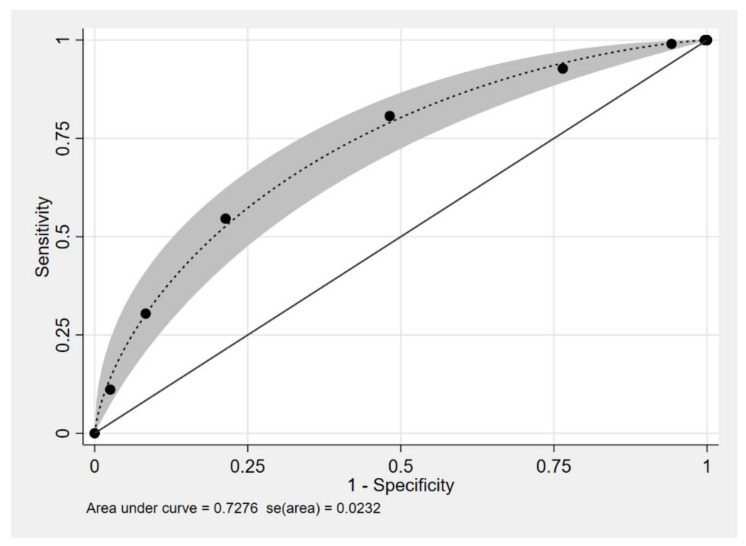
Performance of the clinical model score, area under the receiver operating characteristics (ROC) curve, and 95% confidence band.

Multivariable logistic regression was done. Parameters were selected in the model as defined in method. The regression coefficient, diagnostics odds ratio (dOR), and 95% confidence interval (CI) are shown in Table 2. An age less than 1 month, male gender, term, history of delayed meconium passage, and history of enterocolitis were included in the model. The item scoring scheme for diagnostic parameters for HSCR derived from coefficients is shown in Table 3. The assigned score ranged from 0 to 7 points, which categorized the patients into two risk groups with a cut-off point of 4, as shown in Table 4. The score of low-risk group for being HSCR was 0 to 3 points, while patients with 4 to 7 points were likely to be HSCR. The ROC curve of the clinical score model is shown in Figure 2, which shows the performance of the model. The area under the receiver operating characteristics curve (AuROC) of the clinical score model showed a prediction affinity of 72%, while the full model with five parameters achieved a prediction affinity of 76%.

The Hosmer–Lemeshow goodness of fit statistics test was done for the five parameters model and the clinical score model without statistically significant results (*p*-value = 0.056 and 0.790 respectively). The clinical score model was fit for diagnostic prediction of HSCR in this dataset.

**Figure 3:**
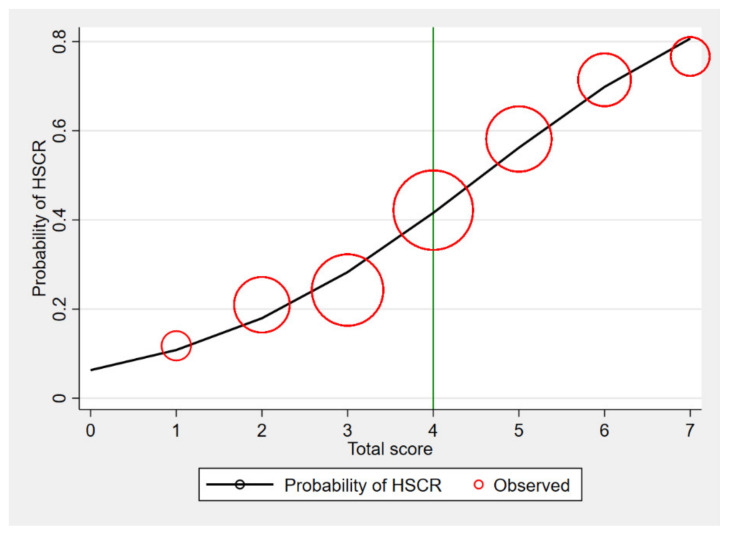
Observed risk (circle) vs. score predicted risk (solid line) of HSCR diagnosis (size of circle represents frequency of HSCR in each score).

The calibration of the model is shown in Figure 3, which compared the observed risk with the score predicted risk of HSCR diagnosis. The score of 4 and more was likely to diagnose of HSCR, and promptly, a referral was suggested to screen the patient to further investigate. The investigations for HSCR, which were contrast enema and rectal suction biopsy, are shown in Table 5.

Internal validation was done by the bootstrapping method, which showed a consistent AuROC 0.763 ± 0.021 with model optimism at 0.008 (range from −0.041 to 0.068).

**Figure 4:**
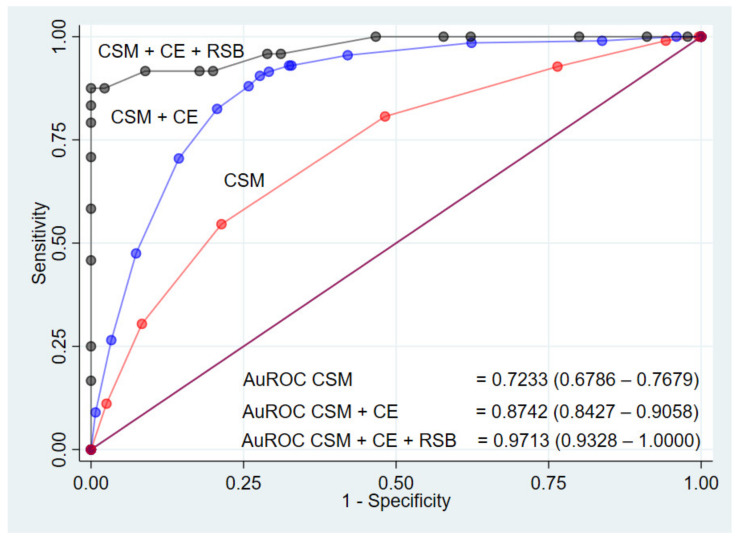
The receiver operating characteristic curve (ROC) for the prediction models for diagnosis of HSCR predicted by clinical score model (CSM), CSM + contrast enema (CE), and CSM + CE + rectal suction biopsy (RSB) (three-curved lines) and a 50% chance prediction (diagonal line).

By the added value concept, the ROC curves with AuROC of the three models are shown in Figure 4. The ability to predict HSCR of clinical score model was 72%. With the addition of contrast enema, the predictive model’s ability to predict HSCR was increased by 15%. Further addition of contrast enema plus rectal suction biopsy made the predictive model’s ability to predict HSCR further improved by 25%, thereby creating a predictive model with a 97% ability to predict HSCR.

Diagnostic values of each parameter are shown in Table 6. Among the clinical score model, contrast enema, and rectal suction biopsy, the parameter that showed the highest sensitivity for diagnosing HSCR was contrast enema and the highest specificity for diagnosing HSCR was the rectal suction biopsy. The clinical score model, which used a cut-off point of 4, was used as a tool for screening with a sensitivity of 80.7% and a specificity of 51.8%. Promptly referring patients for further investigation was advised to avoid delayed diagnosis from clinical observation. However, the patients who were classified as low risk of HSCR should still be monitored and referred when no clinical improvement is seen.

## 4. Discussion

Many conditions could mimic HSCR and confuse clinical practice with a high number of clinically suspected HSCR patients. To make the diagnosis, clinical presentation, physical examinations, and investigations were needed. In the remote hospital, the diagnostic tools available were limited. Some areas have no option for contrast enema or rectal suction biopsy. For this reason, we aspired to find diagnostic clinical parameters for predicting HSCR. In some situations, performing a rectal biopsy in every case might be an over-investigation, as the result from this study showed that more than half of suspected HSCR patients were not diagnosed with HSCR. However, under-investigation could lead to serious complications.

Patients with suspected HSCR were likely to be presented during the neonatal period [22,23,24]. Furthermore, the onset of clinical presentation may vary in the individual through the age of five years old. In this study, the age cut-off point of being lower than one month old can predict HSCR, as patients older than one month usually presented because of other conditions. In our study, these conditions were constipation, anal stenosis, and enterocolitis. However, in patients younger than one month, lactase intolerance was also possible. On the contrary, a previous study [19] of the diagnostic tests for HSCR used a cut-off age at three years old. The pathophysiology of HSCR is congenital in origin. The earliest age from the analysis was chosen, so the cut-off point of age at one month was reasonably considered as our predictor and aid for the early treatment.

The number of male HSCR patients was more than females with a male-to-female ratio of 2.63:1 in our study, which was slightly lower than the normal ratio of HSCR that was 3:1 to 4:1 [23,25,26]. The results from data analysis showed male gender as one of the predictors, which was consistent with the theory.

The weight-for-age percentile in HSCR patients was significantly low compared to non-HSCR patients, as the symptoms of bowel obstruction in HSCR could interrupt growth development and cause insufficient weight gain. However, past studies [27,28] seldom mentioned the weight-for-age percentile as the parameter representing growth in HSCR patients. Moreover, the currently favored definition of failure to thrive, which was weight-for-age less than P5, might not be an appropriate parameter to diagnose HSCR, as HSCR patients were likely to present during the neonatal period when growth was not affected as much.

The proportion of HSCR patients who had a history of delayed passage of meconium at 48 h was significantly higher than non-HSCR patients, which is the same as the results of most studies. Hence, a history of delayed passage of meconium is one of the classic triad symptoms to diagnose HSCR [1,3,7,29]. With the highest specificity (81.5%) among clinical parameters, the history of delayed passage of meconium was an important factor to consider for further investigation. However, there were still a few non-HSCR patients with a history of delayed passage of meconium.

Most of the reports have shown that HSCR patients were likely to occur in term infants. In 2018, the study in California reported the median gestational age at birth was 38 weeks and 6 days [30]. In our study, term infants had very high sensitivity with non-specific parameters for the diagnosis of HSCR.

Enterocolitis was also a presentation and postoperative unfavorable outcome of HSCR. The presence of preoperative enterocolitis was associated with the longer segment and inadequate rectal irrigation [31]. In our study, the presence of enterocolitis was shown as the highest effect size parameter with a dOR of 2.42. However, there was a limitation of missing data. Some of the infants with enterocolitis might not be associated with HSCR.

The results from the contrast enema in this study were consistent with some previous studies [20,21,29], with a sensitivity of 89.00% (83.82–92.98%) and specificity of 72.32% (66.59–77.57%). All the contrast enema signs included in this study showed statistical significance in diagnosing HSCR except jejunization, which can be correlated to the infection. The most common sign in HSCR patients was transitional zone (75.4%), which is the finest clue to diagnose HSCR from contrast enema results [6].

The sensitivity and specificity of RSB were 87.50% (67.64–97.34%) and 98% (89.35–99.95%), respectively, which was consistent with other studies [32,33]. Therefore, RSB is the investigation that can be performed without anesthesia and no rectal scarring left while performing a definite operation, which could be helpful and cost-effective to the patient. However, the RSB needs the equipment and specialized pathologist to interpret the results. So, the clinical diagnosis is still important.

There was a previous clinical prediction model for diagnosis of HSCR [20]. In 2013, the previous score was combined between the clinical risk factors (delayed passage of meconium, age less than 3 years, and male gender) and the investigations that were anorectal manometry, contrast enema, and rectal biopsy. In our clinical score model, we added two more factors that were term infant and previous history of enterocolitis. The age in our study was less than 1 month with the reason stated above. We also obtained a sensitivity of 80.7% without the investigations, which were not available in some areas. This is the screening score to help in early referrals with a shortened time of clinical observation.

The results from data analysis in this study showed high sensitivity of the contrast enema and high specificity of the rectal suction biopsy. Therefore, contrast enema was more appropriate to be used in screening. The result from the contrast enema was also helpful in further surgery to locate the transitional zone. However, due to the high false-positive rate (75/471; 27.7%) and false-negative rate (22/471; 11.0%), subsequently confirming the HSCR diagnosis with rectal suction biopsy was needed. In our study, the addition of contrast enema and rectal suction biopsy improved the area under the ROC curve of clinical parameters only in combination, from 72% to 87% and 97%, respectively. Therefore, the recommendation for the management of patients with suspected HSCR was to screen with clinical parameters first, followed by contrast enema and rectal suction biopsy.

Because this study was retrospective, there were limitations in controlling factors and data collection. The data analysis did not include results from the other two frequently used investigations, which were abdominal plain film and anorectal manometry. The abdominal plain film result was not included because nearly all participants showed bowel dilatation and absence of air in the rectum as the study domain of this study, as well as some of the clinical features such as abdominal distension and vomiting. Anorectal manometry was not included because of recent availability, which also made it difficult to diagnose ultrashort HSCR. A rectal suction biopsy was available in our institute three years ago. Before that, diagnosing HSCR mostly depended on clinical presentations and contrast enema, so some patients had ganglion cells in operative specimens. In the early years, there was also difficulty in differentiating variants of HSCR because of the pathologic understanding of the spectrum of disease.

## 5. Conclusions

Five clinical parameters associated with the diagnosis of HSCR were as follows: age less than one month, male gender, term infant, had history of delayed passing of meconium 48 h after birth, and had history of enterocolitis. Patients with suspected HSCR, who had a clinical score 4–7, had a high probability to be HSCR and were suggested for early referrals for further investigations, which were contrast enema and rectal suction biopsy. In case of a low probability of HSCR, clinical observation and follow-up are still warranted until the symptoms have been resolved. This clinical scoring system can be used as a screening tool to prevent delay diagnosis and complications of HSCR.

## Figures and Tables

**Table 1:** General information, clinical presentations, and physical examination of HSCR and non-HSCR patients in Chiang Mai University Hospital from 2006 to 2020 (all 483 cases).

Characteristics	N	HSCR (*n* = 207)	Non-HSCR (*n* = 276)	*p*-Value
**General Information**				
Age (days) ^a^	483	31.0 (10.0, 153.0)	42.0 (23.0, 99.0)	0.005
Age < 1 month	483	105 (50.7%)	175 (63.4%)	0.007
Age ≥ 1 month	483			
Term	381	147 (88.6%)	167 (77.7%)	0.006
Weight for age (Percentile) ^a^	483	16.6 (3.3, 47.4)	31.0 (6.0, 60.6)	0.026
Gender	483			
Male		150 (72.5%)	139 (50.4%)	<0.001
Female		57 (27.5%)	137 (49.6%)	
Down’s syndrome	349	9 (5.4%)	4 (2.2%)	0.160
**Clinical Presentations**				
History of delayed passage of meconium	308	61 (43.9%)	39 (23.1%)	<0.001
Abdominal distention	450	191 (97.0%)	238 (94.1%)	0.180
Constipation	397	155 (88.1%)	188 (85.1%)	0.460
Bilious vomiting	366	83 (49.4%)	86 (43.4%)	0.290
History of enterocolitis	183	72 (79%)	61 (66%)	0.068
**Physical Examination**				
Failure to thrive	483	139 (72.4%)	199 (76.8%)	0.320
Explosive stool after a rectal examination	383	90 (56.6%)	132 (58.9%)	0.680
Increase rectal sphincter tone on rectal examination	406	112 (66.3%)	159 (67.1%)	0.910

Notes: ^a^ Median (interquartile range). Abbreviation: N, number.

**Table 2:** Regression coefficient, diagnostics odds ratio (dOR), and 95% CI of selected diagnostic parameters derived from logistic regression after multiple imputation.

Diagnostic Parameters	Coefficient	dOR	95% CI of dOR	*p*-Value
Age < 1 month	0.73	2.07	1.37–3.13	0.001
Male gender	0.86	2.37	1.57–3.60	<0.001
Term	0.91	2.47	1.34–4.58	0.004
History of delayed passage of meconium	1.21	3.36	2.03–5.59	<0.001
History of enterocolitis	1.77	5.89	3.01–11.55	<0.001

**Table 3:** Item scoring scheme for diagnostic parameters for HSCR derived from coefficients of select indicators.

Diagnostic Parameters	Coefficients	Transformed Coefficients	Assigned Score
Age at presentation			
<1 month	0.73	1	1
≥1 month	-	-	0
Male gender			
No	-		0
Yes	0.86	1.18	1
Term			
No	-	-	0
Yes	0.91	1.25	1
History of delayed passage of meconium			
No	-	-	0
Yes	1.21	1.65	2
History of enterocolitis			
No	-	-	0
Yes	1.77	2.42	2

**Table 4:** Distribution of risk of diagnosis of HSCR, LR+, and 95% CI of LR+.

Risk Level	HSCR N (%)	Non-HSCR N (%)	PPV (%)	LR+	95% CI of LR+	*p*-Value
Low (score < 4; 0–3)	40 (21.9)	143 (78.1)	21.9	0.37	0.28–0.50	<0.001
High (score ≥ 4; 4–7)	167 (55.7)	133 (44.3)	55.7	1.67	1.46–1.92	<0.001

Abbreviation: N, number; PPV, positive predictive value; LR+, likelihood ratio of positive; CI, confidence interval.

**Table 5:** Results of investigation of HSCR and non-HSCR patients in Chiang Mai University Hospital from 2006 to 2020.

Characteristics	N	HSCR (*n* = 207)	Non-HSCR (*n* = 276)	*p*-Value
**Investigation**				
Contrast enema	471			
Transitional zone		156 (75.4%)	62 (22.5%)	<0.001
Jejunization		156 (75.4%)	62 (22.5%)	0.089
Saw-tooth appearance		33 (15.9%)	17 (6.2%)	<0.001
Reverse rectosigmoid ratio (>1)		87 (42.0%)	55 (19.9%)	<0.001
Delayed evacuation in 24 h		114 (55.1%)	122 (44.2%)	0.021
Contrast enema result	471	178 (89.0%)	75 (27.7%)	<0.001
Rectal suction biopsy	74			
Positive for GG or calretinin positive (Not HSCR)		3 (13%)	49 (98%)	<0.001
Negative for GG or calretinin negative (Diagnose HSCR)		21 (88%)	1 (2%)	

Abbreviation: GG, ganglion cells; HSCR, Hirschsprung’s disease; N, number.

**Table 6:** Sensitivity, specificity, PPV, and NPV.

Parameters	N	Sensitivity	Specificity	PPV	NPV
Age < 1 month	483	49.3%	63.4%	50.2%	62.5%
Male gender	483	72.5%	49.6%	51.9%	70.6%
Term	483	90.8%	19.6%	45.9%	74.0%
History of delayed passage of meconium	483	41.1%	81.5%	62.5%	64.8%
History of enterocolitis	483	87.0%	26.8%	47.1%	73.3%
Clinical score model cut-off point ≥ 4	483	80.7%	51.8%	55.7%	78.1%
Contrast enema	471	89.0%	72.3%	70.4%	89.9%
Rectal suction biopsy	74	87.5%	98.0%	95.5%	94.2%

Abbreviation: N, number; NPV, negative predictive value; PPV, positive predictive value.

## Data Availability

The datasets used during the current study are available from the corresponding author on reasonable request.
